# Intention to voluntary blood donation among private higher education students, Jimma town, Oromia, Ethiopia: Application of the theory of planned behaviour

**DOI:** 10.1371/journal.pone.0247040

**Published:** 2021-03-02

**Authors:** Abiot Aschale, Diriba Fufa, Tilahun Kekeba, Zewdie Birhanu

**Affiliations:** 1 Department of Public Health, College of Health Sciences Mizan, Tepi University, Mizan, Ethiopia; 2 Faculty of Public Health, Department of Monitoring and Evaluation, Jimma University, Jimma, Ethiopia; 3 Department of Paediatrics, Institute of Health, Jimma University, Jimma, Ethiopia; 4 Faculty of Public Health, Department of Health, Behavior, and Society, Jimma University, Jimma, Ethiopia; Endeavour College of Natural Health, AUSTRALIA

## Abstract

**Background:**

Blood is an important and crucial component in the management of patients presenting with severe accident injuries, surgical conditions, malignancies, pregnancy-related complications, and other medical conditions.

**Objectives:**

To assess intention to voluntary blood donation among private higher education students in Jimma Town, South West Ethiopia, 2019.

**Methods:**

Institution-based cross-sectional study with quantitative methods was conducted in private higher education students in Jimma town. A multistage sampling technique was used to recruit study participants. First, a simple random sampling technique was used to select departments in each private higher education institution. Seven departments were included in the study and after proportionally allocated in each department, a total of 595 were participated in the study, producing a response rate of 98%. The data was collected using self-administered structured questioners with 3 trained data collectors. Multivariable linear regression analysis was done to assess association between the independent variables and dependent variable.

**Results:**

The mean score for intention of the respondents to donate blood voluntarily was 15.41 out of 25 with standard deviation of 4.42.The TPB variables explained 61.3% of the variance of intention to donate blood. Direct perceived behavioral control (β = 0.745, P < 0.001), direct attitude (B = 0.295, P<0.001) and direct subjective norm (β = 0.131, P< 0.001) were significant predictors of the intention.

**Conclusion:**

Respondents’ intentions are mainly determined by perceived barriers and, subjective norms, the attitude of respondents towards voluntary blood donation.

## Introduction

Blood is universally recognized as the most precious element that sustains life. It is an important and crucial component in the management of patients presenting with severe accident injuries, surgical conditions, malignancies, pregnancy-related complications, and other medical conditions [1]. World Health Organization report on blood donation indicates the critical shortcomings in blood transfusion, especially in the low-income countries. Of the 112.5 million blood donations collected globally, approximately half of these are collected in high-income countries, home to only 19% of the world’s population [2].

Every day, about 800 women die from pregnancy or childbirth-related complications. Severe bleeding during delivery and after childbirth is a major cause of mortality, morbidity, and long-term disability, therefore, ensuring timely access to safe blood and blood products is essential for all countries as part of a comprehensive approach to prevent maternal deaths due to severe bleeding [3]. In Africa, there is a high demand for blood transfusion due to bleeding related to pregnancy and childbirth, high prevalence of malaria with the attendant complication of severe malarial anemia, high rates of road traffic accidents and other types of injury as well as other indications for a blood transfusion but 38countries of the region collected fewer than 10 donations per 1000 people and more than 50% of the blood supply is still dependent on family members and paid blood donors [4]. In the context of our country, Ethiopia is categorized as one of the countries with a very low blood donation rate which is 0.6per thousand populations next to Nigeria [5].

According to the Ethiopia Health sector transformation plan (HSTP) report of 2015/16, it was planned to collect 202,000 units of blood but the collected was 169, 744 units of blood [6].

The essence of TPB is that a person’s readiness to perform a specific behavior. According to the theory, the origin of both intention and actual behavior are from an individual’s attitudes, subjective norms. In an attempt to predict an individual intention and actual behavior, TPB spells out three main factors which are an Attitude toward Behavior (ATB), subjective norms (SN), and Perceived Behavioral Control [7].

Attitude towards Behavior (ATB). The first construct of TPB is the ATB where it is defined as an overall evaluation of one’s behavior [8]. The construct indicates the degree of performance behavior is valued. Usually, ATB is accessed through behavioral belief where it links the interesting behavior with the expected outcomes. This is because behavioral belief represents an individual’s self-reported outcome evaluation.

Subjective Norms (SN). By definition, norms are defined as something that reflects the feeling of personal responsibility to perform a behavior [9], in which it implies an individual social pressure or influence to involve in a specific behavior. TPB explained SN as a construct that determined by normative beliefs which is the expectation that complies with the motivation that an individual in common, where a person like to perform something that other people such as their family members, friends, supervisor or the society like to perform since they perceived the behavior is appropriate to perform. This is where, a person will be using information about others to adjust their behavior and as a result, he or she will perform the same behavior and perceived it as a common behavior in the group.

Perceived Behavioral Control (PBC). PBC is an individual perception’ of their ability to perform a behavior [10]. It is based on the individual feeling of having control over their behavior. Usually, PBC will tend to exist when a person estimates the level of difficulty for him or her to perform a specific behavior. This is where an individual control belief will influence his or her actual behavior. At the time a person had a perceived factor that presents in common which may facilitate the performance of actual behavior [11]. This is because PBC will assist the prediction of one’s intention to perform since the factors may influence one’s decision. As for an example, a person may have a high willingness to donate his or her blood if he or she is confident with his or her ability to survive after the blood transfusion process. While, those who feel that blood donation is not in their control, he or will tend to not engage with the actual behavior [10].

Generally, according to previous researches, the Theory of planned behavior (TPB) is a good predictor of behavioral intention to blood donation [12,21]. All three components of TPB have very sufficient contribution in predicting the population’s intention to donate blood [12]. However, little is known about the applicability of the TPB in predicting voluntary blood donation in Ethiopia. Therefore, this study aims to assess the intention of students towards blood donation based on the theory of planned behavior.

According to Jimma blood bank unpublished report on 2018, the prevalence of blood donation among private higher education institutions was lower (36%) than governmental institutions which was 63% [13]. So this study focused on private institutions rather than governmental institutions.

## Methods

### Study area and period

The study was conducted in Jimma town which is located 354 Km Southwest of Addis Ababa. The town has an altitude of 1750-2000m above sea level, a temperature range of 20–30 oC. According to the national census of 2007, the projected total population of the town is 174, 396 (86,326 males and 88,070 females). It has two public hospitals, one private hospital and it has also one blood bank. There are a total of three private higher education institutions in the town. There are about 10500 students in these institutions. The research took place among 3 private higher education institutions in Jimma town. All of the participants were 18 years old and above. Both male and female students participated in the study. The study was conducted from March to April 1, 2019.

### Study design

An institution-based cross-sectional study with quantitative methods was conducted in selected higher education students in Jimma Town.

#### Study population

The study population were sampled higher education students in Jimma town.

Private higher education students who were seriously ill, not present at the time of data collection and below 18 years were excluded from the study.

#### Sample size determination

The sample size was determined using a single population proportion using the proportion of the intention to voluntary blood donation 50% since there was no similar previous study in similar area in Ethiopia. And with 95% confidence interval and 5% margin of error

$$n=\frac{\left(Za/2)2p(1-p\right)}{d^{2}}=(1.96{)}^{2}(0.5)(0.5)/0.05(0.05)=384$$

Where, n = required sample size

*Za*/2 = critical value for normal distribution at 95% confidence interval which equals 1.96 (*Z* value at alpha = 0.05) p- The proportion of population intended to voluntary blood donation. Considering design effect 1.5, and adding a non-response rate 5%, Nf = 604.

#### Sampling technique and procedure

A multistage sampling technique was used to recruit study participants. First, a simple random sampling technique was used to select departments in each private higher education institution. Those who are randomly selected departments in each institution were 30% and above out of a total number of departments in each institution. In Jimma town, there are three private higher education institutions. Those are Rift Valley University, Dandi Boru, and Afro Canada colleges. Rift valley University has departments of Accounting, ICT, Midwifery, Pharmacy, Nursing, Public health officer, Business Management administration. Dandi Bouru College also has departments of veterinary, ICT, Nursing, Accounting, management. Afro Canada college has two departments Accounting, ICT. First among three institutions by using simple random sampling participants were selected from4 departments (from Management 152 participants, Economics76 participants, Public Health 46 participants, Pharmacy 87 participants) in Rift Valley University and 2 departments (veterinary120 participants, ICT 69 participants) in Dandi Bouru college,1 department (accounting 54 participants) in Afro Canada by considering a list of departments as the sampling frame. The sample size was proportionately allocated from each selected department based on their academic year. Finally, by using the students’ name list from the registrar as the sampling frame, the respondents were selected by a simple random sampling method for the self-administered questionnaire.

#### Data collection instrument

The questionnaire was developed by review of related literatures TPB Question (for direct measures) [8] and through elicitation study (for indirect measures). The questionnaire was structured into 11-sections (intention, direct and indirect attitude, subjective norm, and perceived behavioral control, past behavioral practice, socio-demographic characteristics and knowledge). A five option Likert scale was used for direct and indirect measures. The questionnaire was prepared in English and translated to the local language (Afan Oromo, Amharic) for appropriateness and easiness. Then it was translated back to English to check for consistency of meaning. Language experts in all cases did a translation of the questionnaire. For the elicitation study, 25 participants were recruited by the judgmental (purposive) sampling technique. The participants for IDI were selected from each selected college. The result from the qualitative study was used to develop a tool for the indirect measurements of the constructs of TPB (an indirect measurement of Attitude, subjective norm, and PBC) by revealing the silent beliefs of each construct.

Semi-structured IDI guideline was prepared based on the predictive constructs of the TPB model (attitude; subjective norm; and perceived behavioral control).

Elicitation was done using semi-structured IDI guideline that is designed by reviewing TPB guidelines and modifying for purpose of this study. The quantitative data were collected by using self-administered structured questioners with 3 trained data collectors and 3 supervisors for 10days.

To assure the quality of data; Pretest was done by asking 5% of the sampled population to respond to the questionnaire. The data collection questionnaire first was prepared in English and then was translated to local language (Afan Oromo, Amharic) and back to English for checking language consistency by a different person with excellent Afan Oromo, Amharic, and English speaking skill.

#### Data processing and analysis

**Fig 1:**
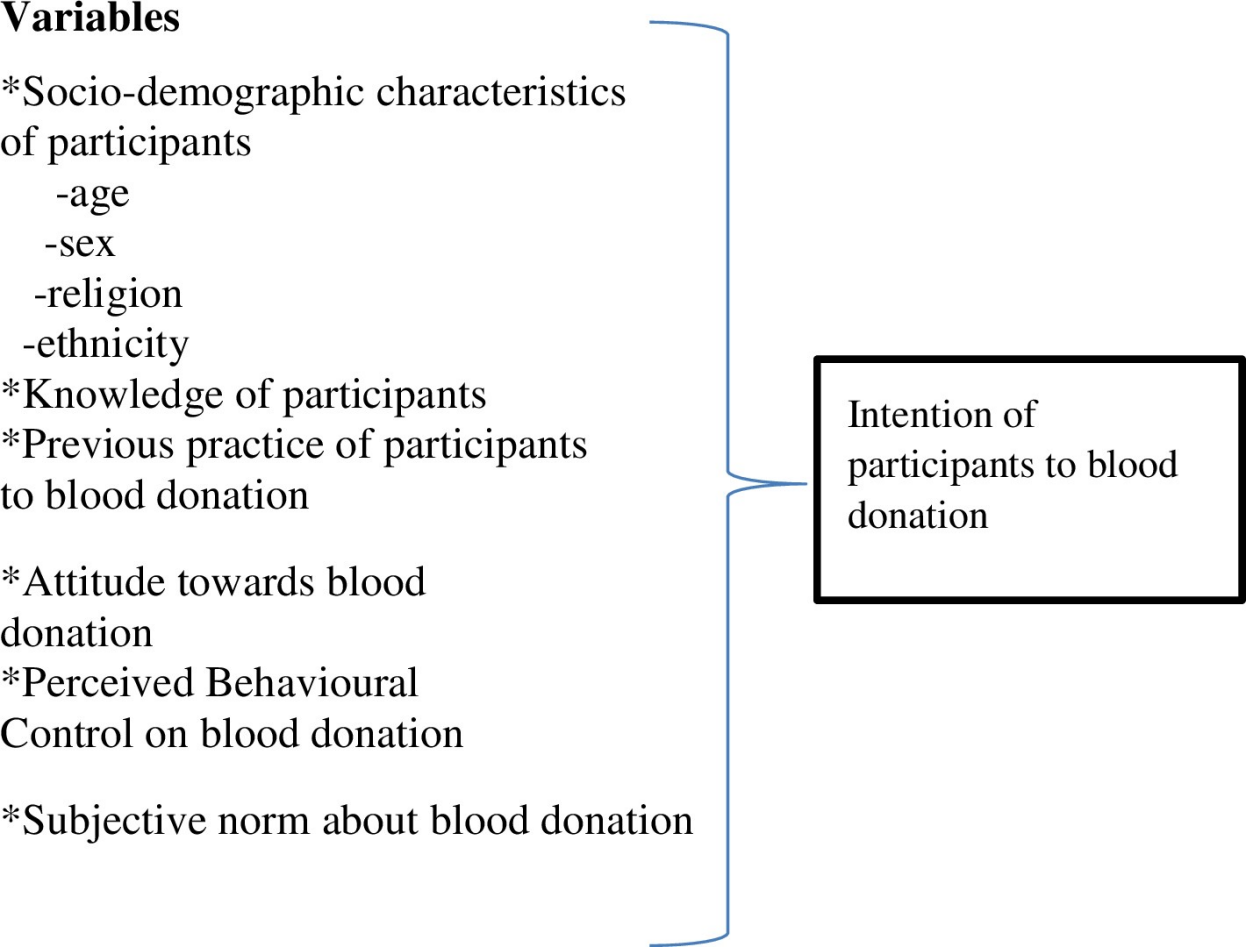
Showing dependent and independent variables.

Before the use of the instrument, the reliability correlation coefficients for the TPB constructs were checked to assess the validity of the tool. Cronbach’s alpha was used to assess the internal consistency of the measurement scales in the survey instrument (i.e. attitude, subjective norms, perceived behavioral control, intention) using the entire sample. Cronbach’s coefficient Alpha at 0.7 or higher score was accepted for all the scales. Data was checked for its completeness, by the data collectors, the supervisors and the investigator on the field, and during data entry then it was edited, coded, and entered by Epidata version 4.4.1 and exported to SPSS-20 for the analysis. Descriptive statistical measures like frequency, distribution, the mean and standard deviation were done. Simple linear regression analysis was done to assess the association between independent variables and intention as shown in Fig 1.

All variables at a p-value less than 0.25 in simple linear regression analysis were entered into multiple linear regressions to identify the independent predictors of intention. Correlational analysis was done between the direct and the indirect measurements of TPB constructs to identify the direction and relationship between them. A p-value less than 0.05 is considered to indicate a significant association. Using a multiple regression procedure direct measures of TPB constructs attitude, subjective norm, and perceived behavioral control as the predictor variables and the intention was entered as the dependent variable. Indirect measures of each behavioral belief were multiplied (weighted) by the score for the relevant outcome evaluation to create a new variable that represents the weighted score for each behavioral belief. Similarly, each normative belief is weighted by the score for motivation to comply and each control belief by the score representing the influence of the control belief. Then the weighted beliefs were summed up to create a composite score for attitude, subjective norm, and perceived behavioral control respectively.

#### Ethical considerations

Ethical approval was obtained from the Institutional Review Board of Jimma University, Institute of health. Official letter of cooperation was taken from the Institute of Health of Jimma University to Rift Valley University, Dandi Bouru College, and Afro Canadian College. Letter of the permission was obtained from Rift Valley University, Dandi Bouru College, and Afro Canadian College. Written informed consent was obtained from participants after thoroughly explaining the objectives and benefits of a study. To ensure confidentiality and anonymity, any personal identifying information on participants was not collected.

## Results

### Socio-demographic characteristics of respondents

Five hundred ninety-five participants were participated in the study, producing a response rate of 98%. Table 1 contains detailed demographic characteristics of the participants. Consequently, of the participants, 330 (55.5%) were female and the mean age of respondents was 20.46 (SD = ±2.32) years. The majority of the participants, 228 (38.3%) were followers of Orthodox Christianity followed by Protestants 162 (27.2%) and Islam 156 (26.2%). Three hundred twenty-eight 328 (55.1%,) of the respondents were Oromo followed by Amhara 90 (15%).

**Table 1:** Socio-demographic characteristics of respondents 2019 (n = 595).

Variable	Frequency	Percent
Sex		
Male	265	44.5
Female	330	55.5
Age at completed years		
18–20	371	62.4
21–23	166	27 .9
24–26	43	7.2
≥27	15	2.5
**Religion of participants**		
** Islam**	156	26.2
**Christian**	439	73.8
-Orthodox	228	38.3
-Protestant	162	27.2
-Catholic	24	4.0
Others*	25	4.2
**Ethnicity**		
Oromo	328	55.1
Amhara	90	15.1
Dawuro	40	6.7
Gurage	18	3.0
Tigre	7	1.2
Others**	112	18.8
Department		
Accounting	54	9.1
Economics	76	12.8
Public Health	46	7.7
Information technology	69	11.6
Business management	143	24.0
Pharmacy	87	14.6
Veterinary	120	20.2
**Year of study**		
1^st^	360	60.5
2^nd^	60	10.1
3^rd^	160	26.9
4^th^	15	2.5
**Previous residence**		
Urban	315	52.9
Rural	280	47.1
Total	595	100

* = Joba witness, wakkafeta.

** = Sheka, Kaffa, Yem.

### Knowledge of voluntary blood donation

Respondents’ response to knowledge questions is presented in Table 2. Regarding the minimum age-eligible for blood donation, 292 (49.1%) of the respondents mentioned that 18 years while, 303 (50.9%) did not know the minimum age to donate blood. Additionally, regarding the minimum weight eligible for blood donation, 318 (53.4%) of the respondents replied that they did not know the eligible weight required for blood donation. Only 277 (46.6%) of the respondents mentioned that 45 to 50 kg is eligible for blood donation. On the other hand, 257 (43.2%) of the respondents reported that one can donate blood every 3 months. The overall mean knowledge score is 2.21 (SD ±1.349) with a range of possible values of 0–5.

**Table 2:** Respondents knowledge on voluntary blood donation among students attending private colleges in Jimma town, Oromia, Ethiopia.

Variable/question	Response	Number	Percent
Knowing the minimum age eligibility for blood donation	Yes (18 Yrs.)	292	49.1%
	Don’t know	303	50.9%
Knowing the minimum weight eligibility for blood donation	45–50 kg	277	46.6
	Don’t know	318	53.4
Knowing the time interval between donations	Yes (3months)	257	43.2
	Don’t know	338	56.8
Knowing the amount of blood donated once per milliliter	Yes (350–450 ml)	29	4.9
	No	566	95.1

### Past behavioral practice on voluntary blood donation

Participants’ response regarding the past behavioral practice of VBD is shown in Table 3.

**Table 3:** Past behavioral practice on voluntary blood donation.

Question/variable	Frequency	Percent
Have you ever requested to donate blood		
Yes	466	78.3
No	129	22
Have you accepted the donation		
Yes	137	29.2
No	332	70.8
Ever donated blood in the past		
Yes	123	20.7
No	472	79.3
How many times did you donate		
1–2 times	102	82.9
3 to 4 times	15	12.2
> 4 times	6	4.9
Waiting time after last donation		
1–3 months	31	25.2
4–6 month	29	23.6
6 months	63	51.2
Purpose of donation		
For unspecified person/Voluntary/	103	83.7
For family,relative, friends	20	16.3

Only 123 (20.7%) of respondents donated blood in the past and the remaining 472 (79.3%) of respondents did not donate blood previously. Of those who donated blood, 102 (82.9%) of respondents donated one to two times, 15 (12.2%) of respondents donated three to four times and 6 (4.9%) of respondents donated greater than four times. From those previously donated respondents, 31 (25.2%) waited 1–3 months while others waited for greater than 3 months.

### The intention of respondents to donate blood voluntarily

Descriptive findings for each intention measuring showed that 40 (6.7%) of the respondents strongly agree to the statement “I plan to donate blood in the next six months” and 251 (42.2%) of the respondents did not have any decision (undecided) on this statement. The mean score for the intention of the respondents to donate blood voluntarily was 15.41 out of 25 with standard deviation of 4.42.

### Direct TPB model components

The direct measures towards voluntary blood donation included attitude, subjective norms, and perceived behavioral control is shown in Table 4. Concerning the frequency of the direct attitude, 170 (28.6%) of respondents strongly agreed, 308 (51.8%) agreed, 30 (5%) disagreed, 8 (1.3% strongly disagreed and 79 (13.3%) of respondents had a neutral response on the item “donating blood is pleasant”. On the other hand, 248 (41.7) of respondents strongly disagreed, 297 (49.9) disagreed, 11 (1.8%) agreed, 2 (0.3%) strongly agree and the remaining 7 (6.2%) of respondents were undecided on the statement “donating blood is the wrong thing to do”.

**Table 4:** Frequency of direct TBP constructs among respondents in Jimma Town, Oromia, Ethiopia.

Direct measure items	Strongly disagree	Disagree	Undecided	Agree	Strongly agree
	No (%)	No (%)	No (%	No (%)	No (%)
Attitude					
I think donating blood is pleasant	8 (1.3)	30 (5)	79 (13.3)	308 (51.8)	170 (28.6)
I think donating blood is the wrong thing to do	248 (41.7)	297 (49.9)	37 (6.2)	11 (1.8)	2 (0.3)
I think donating blood is a good idea.	2 (0.3)	15 (2.5)	35 (5.9)	356 (59.8)	187 (31.4)
I think donating blood is unnecessary	268 (45)	288 (48.4)	28 (4.7)	11 (1.8)	0
Direct subjective norm					
Most important people to me think that I should NOT donate blood	76 (12.8)	187 (31.4)	94 (15.8)	206 (34.6)	32 (5.4)
Most people who are important to me approve of my blood donation	36 (6.1)	196 (32.9)	152 (25.5)	168 (28.2)	43 (7.2)
I feel under social pressure to donate blood	81 (13.6)	246 (41.3)	117 (19.7)	127 (21.3)	24 (4)
I am socially expected to donate blood	30 (5)	139 (23.4)	176 (29.6)	211 (35.5)	39 (6.6)
Perceived behavioral control					
People who are important to me want me to donate blood	42 (7.1)	188 (31.6)	167 (28.1)	160 (26.9)	38 (6.4)
I am confident I can donate blood in the next six months.	46 (7.7)	149 (25)	208 (35)	136 (22.9)	36 (9.4)
I am too weak to donate blood in the next six months	87 (14.6)	237 (39.8)	105 (17.6)	143 (24)	23 (3.9)
It is easy to donate blood	84 (14.1)	249 (41.8)	78 (13.1)	140 (23.5)	44 (7.4)
I do not have enough blood to donate	69 (11.6)	167 (28.1)	97 (16.3)	220 (37)	42 (7.1)
Donating blood for the next six months is up to me	36 (6.1)	141 (23.7)	201 (33.8)	164 (27.5)	53 (8.9)

### Summary statistics for direct components of TPB

Descriptive statistics analysis was done to compute the mean score for direct TPB components. Direct attitude, subjective norm, and perceived behavioral control had a mean score of mean (±SD) score of 16.88 (±2.36), 15.57 (±3.5), and 14.80 (±3.80) respectively. The mean score of intention was 15.4 out of 25 with a standard deviation of 4.4.

### Indirect TPB model components

#### A. Indirect attitude measurement items

The behavioral belief result showed that 312 (52.4%) agree, 194 (32%) strongly agree on the item “if I donate blood I save patients life”. On the other hand, 224 (37%) of respondents agree and 119 (20%) of respondents strongly agree on the item “when I donate blood I become anemic”.

In the evaluation outcome result 338 (56. 8%) of respondents agree, 130 (21.8%) strongly agree on the item “having headache is undesirable to me”. Additionally, 271 (45.5%) of respondents agree and 239 (40.2%) strongly agree on the item “becoming anemic is bad to me”.

#### B. Indirect subjective norm

In the normative belief result, 88 (14.8%) of respondents strongly disagree, 244 (41%) disagree on the statement “my family think that I should donate blood”.

The motivation to comply for normative belief result showed that 245 (41.2%) respondents agree and 44 (7.4%) of respondents strongly agree with the statement “I want to do what my family thinks I should do”. On the other hand, 181 (30.4%) of the respondents agree and 34 (5.7%) of respondents strongly agree with the statement “I want to do what my friend thinks I should do”.

#### C. Indirect perceived behavioral control on voluntary blood donation

The control belief result showed that 100 (16.8%) of the respondents strongly disagree, 169 (28.4%) disagree, 212 (35.6%) agree, 73 (12.3%) strongly agree and the remaining 41 (6.9%) of the respondents had a neutral idea on item fear of pain from injections makes me be deferred from donating blood.

The power of control beliefs result showed that 213 (35.1%) respondents agree and 44 (7.4%) strongly agree with the statement “lack of balanced diet prevents me from donating blood”.

### Summary statistics for indirect measures of TPB

Descriptive statistics analysis was done to measure the mean score of indirect TPB components as shown in **Table 5**. Indirect attitude had a mean score of 97.75 (SD = ±29.97). Similarly, indirect PBC and indirect subjective norm had a mean scores of 55.6 (SD = ±21.87), 53.97 (SD = ±26.2) respectively.

**Table 5:** Descriptive statistics for the indirect components of the theory of planned behavior model among private college students in Jimma town, Oromia, Ethiopia, 2019 (n = 595).

Components	No of items	Min. value	Max. value	Mean (%)	SD
Behavioral belief (BB)	10	15	50	31.2 (62.4)	7.51
Outcome evaluation (OE)	10	17	46	27.7 (55.4)	4.86
Indirect attitude = £ (BB*OE)	10	28	210	97.8 (39)	29.97
Normative belief (NB)	6	6	30	16.9 (56.3)	5.27
Motivation to comply (MC)	6	6	30	18.7 (62.3)	4.50
Indirect subjective norm (IDSN) = = £ (NB*MC)	6	6	150	53.2 (35.5)	26.2
Control belief (CB)	5	5	25	16.1 (64.2)	4.06
Perceived power (PP)	5	7	25	16.2 (64.8)	3.34
Indirect PBC (IDPBC) = = £ (CB*PP)	5	9	125	55.6 (44.5)	21.87

### Correlation analysis

#### Correlation between direct TPB constructs and intention to voluntary blood donation

Table 6 shows the correlations between direct TPB constructs and intention to voluntary blood donation. It indicates that perceived behavioral control has a strong relation with the intention to voluntary blood donation (r = 0.764), while subjective norm (r = 0.477) and Attitude (r = 0.458) have a moderate correlation with the intention to voluntary blood donation.

**Table 6:** Correlations between direct TPB constructs and intention to voluntary blood donation.

Measures	Pearson correlation (r)	Attitude	SN	DPBC	Intention
Attitude	R	1			
SN	R	.311^**^	1		
PBC	R	.414^**^	.500^**^	1	
Intention	R	.458^**^	.477^**^	.764^**^	1

**Correlation is significant at the 0.01 level (2-tailed).

Correlation of direct and indirect TPB constructs.

Table 7 shows a correlation between direct and indirect TPB constructs.

**Table 7:** Bivariate correlation (Pearson’s r) b/n direct and indirect measures of TPB model among private college students in Jimma town, Oromia, South West Ethiopia, and March 2019.

Measures	Pearson correlation (r)	DAtt	Direct SN	DPBC	InDAtt	InDSN	InDPBC
DAtt	r	1					
DSN	r	.311^**^	1				
DPBC	r	.414^**^	.500^**^	1			
InDAtt	r	.503^**^	.453^**^	.580^**^	1		
InDSN	r	.253^**^	.529^**^	.415^**^	.374^**^	1	
InDPBC	r	.345^**^	. 319^**^	.450^**^	.510^**^	.301^**^	1

**Correlation is significant at the 0.01 level (2-tailed).

DAtt = direct attitude, DSN = direct subjective norm,DPBC = direct percived behavioural control,InDAtt = indirect attitude,InDSN = indirect subjective norm,InDPBC = indirect perceived behavioural control.

The correlation between direct and indirect measures of TPB in the context of voluntary blood donation is ranged from 0. 253 to 0.580 suggesting that the direct measures are independent predictors of voluntary blood donation.

### Linear regression analysis

Of the external variables, knowledge (B = 1.084, 95% CI = 0.83,1.33,P< 0.001), Ever donated blood in the past (B = 3.99, p< 0.001, 95%CI = 3.173,4.812), previous urban residence (B = 1.65, P< 0.001, 95% CI = 0.95, 2.35, P< 0.001) had statically significant bivariate regression with intention while other socio demographic variables like religion, sex, age, field of study and other categorical variables were not significantly associated. From TPB variables direct attitude (B = 0.854, P< 0.001, 95% CI = 0.720, 0.987), DSN (B = 0.597, P< 0.001, 95% CI = 0.508, 0.686), DPBC (B = 0.881, P< 0.001, 95%CI = 0.821, 0.941), 95% CI = 0.062, 0.086) were regressed.

#### Multivariable linear regression analysis of all variables

Candidate variables from simple linear regression analysis were entered to multiple linear regressions analysis as indicated in Table 8. Of the variables entered into the model using the stepwise method, only three variables remain significant and retained in the final model. Accordingly, from multiple linear regression analysis direct attitude (B = 0.295, P< 0.001), direct subjective norm (B = 0.131, p<0.001), direct perceived behavioral control (B = 0.745, P< 0.001). This means a positive unit change in attitude towards the advantage of voluntary blood donation; intention to donate blood will be increased by 0.745 and provided that other variables kept constant. Similarly, for a positive unit change in subjective norm (positive social pressure to donate blood), intention to donate blood will be increased by 0.131units provided that other variables are kept constant. Additionally, a positive unite change in perceived behavioral control factors, intention to donate blood will be increased by 0.745 units provided that other variables are kept constant.

**Table 8:** Predictors of intention to voluntary blood donation among private college students in Jimma town, Jimma town, Oromia, Ethiopia (n = 595) 2019.

Model	Unstandardized Coefficients	Sig.	95%CI		VIF
	B		Lower Bound	Upper Bound	
DPBC	.745	.000	.675	.815	*1*.*48*
DAtt	.295	.000	0.191	0.398	*1*.*228*
DSN	.131	.000	0.058	0.204	*1*.*357*

Adjusted r^2^ = 61.3.

DPBC **=** direct percived behavioural control,DAtt = direct attitude,DSN = direct subjective norm,VIF = variance infletion factor.

## Discussion

This study describes the intention to voluntary blood donation among college or university students. Among the objectives of the study, one was identifying socio-demographic characteristics associated with intention to VBD. However, the results of this study revealed no significant socio-demographic predictor variable. This implies that the age, the religion of students does not determine their intention to donate blood. In contrast to this, the study conducted in Spain, India, Botswana indicated that people of younger age had more intention to donate blood than elders [14–17]. Also study in Dire Dawa indicated a high intention of blood donation with the age group 18 to 35 [18]. This difference may be due to the age characteristics of respondents. In the current study, participants are almost within a similar age category and all participants are below age 35. Sex was also significantly associated with intention to voluntary blood donation in studies conducted in different areas [17–20]. Regarding knowledge of respondents on eligibility criteria for blood donation, from a total of the respondents, 303 (51.9%) and 318 (53.4%) didn’t know the minimum age and weight limit to donate blood respectively. This study shows that more than half of the students did not have awareness about blood donation. This finding is higher than the study conducted in Addis Ababa University health science students in which 14.3% and 9.6% of students did not know the age and weight limit required for blood donation respectively [16]. The difference may be due to respondent’s discipline. The previous study included only on health-Science students who are supposed to be knowledgeable but in this study, both health and non-health students were included in the study.

Majority of the respondents with a past history of blood donation 83.7% donated for the unspecified persons (voluntarily) while 20 (16.3%) of respondents donated for family, relatives, and friends. This finding is higher than the study conducted in Tigray only 19.8% donate voluntarily and about 82% of respondents donate for the replacement (family, paid) [21]. The difference may be due to population characteristics and the time of the study. Even if one hundred twenty-three (20.6%) of respondents have previously donated blood but their past behavioral practice does not have a significant effect on the intention to future donation. This finding may be due to low satisfaction with health professionals or lack of positive reinforcement after voluntary blood donation. The mean intention of the respondents to donate blood voluntarily was higher than the study conducted in Dire Dawa [18]. The difference may be due to educational status. In this study, all participants are educated and are supposed to be relatively less affected by misconceptions about voluntary blood donation than the population in the previous study. Concerning the prediction of TPB constructs in this study, intention to voluntary blood donation among respondents was mostly influenced by perceived behavioral, attitude and subjective norm to voluntary blood donation. It shows that respondents with lower perceived behavioral control, attitude, and subjective norm expressed weaker intentions to blood donation. Direct perceived behavioral control had strong association with intention to blood donation than direct attitude and direct subjective norm.This finding is in agreement with a study conducted in Dire Dawa that has the strongest relationship with behavioral intention was PBC [18]. Also, it is supported by research conducted in Malaysia which suggests that a person may have a high willingness to donate his or her blood if he or she is confident with his or her ability to survive after the blood donation [12]. In contrast to this, the study conducted previously found that perceived behavioral control, on its own, did not have a significant effect on blood donation [21]. This indicates that other TPB constructs attitude, subjective norms have a great role in determining the intention of students to blood donation. The direct attitude was also significantly associated with intention to blood donation. This implies that participants with a negative attitude towards blood donation have not a willingness to donate blood whereas students with a positive attitude will have the willingness to donate blood. This finding is similar to a study done in Tigray in which attitude was significantly associated with intention to blood donation [17]. Respondents’ attitude on blood donation was varied from 28 to 210 with a mean score of 53.2 and standard deviation of ±29.97 which is low. This is due to the majority of the participants in this study believe that “blood donation causes anemia, weight loss, headache and fainting”.The indirect subjective norm finding on blood donation varied from 6 to 150 with a mean score of 53.2 and standard deviation of ±26.2 (Table 6). This finding suggests that students are influenced by significant others like families, classmates, teachers, not to donate blood. This study agrees with previous studies in which the influence of family and significant others on blood donation was demonstrated [12,21]. Therfore, interventions to improve blood donation intention should also target those important others (families, class mates,teachers) as a whole rather than focusing only on students who are eligible for blood donation. If their important others are engaged in blood donation practice, eligible individuals are more likely to participate in blood donation.

### Strengths and limitation

This is the first study to determine the intention to Voluntary blood donation among higher education students based on TPB in Ethiopia. Although the findings have meaningful implications in determining the predictors of the behavioral intention of students towards voluntary blood donation, we studied intention rather than actual behavior. The study used a self-administered questionnaire and self-reported method may have some problems such as over-reporting desirable beliefs and under-reporting undesirable beliefs and behaviors.

### Conclusion and recommendation

Perceived behavioral control, subjective norm, attitude, were significantly associated with intention to blood donation. The result of this study implies that respondents’ intentions are mainly determined by perceived barriers and obstacles, subjective norms, attitude of individuals. The mean intention of the respondents to donate blood voluntarily was low.This suggests that there are beliefs and other knowledge related factors that lead students not to donate blood. Therefore, health education concerning the importance and need of blood donations should be given to all students in higher education institutions; as an education program to increase awareness of students towards blood donation. Educational inteventions should be targeted at changing negative attitudes on voluntary blood donation to enhance the intention of students to voluntary blood donation.

## Abbreviations

TPB: Theory of planned behavior
WHO: World Health Organization
SPSS: Stastical package for Social Science
IDI: Indepth-interview
SD: standard devation

## References

[1] WHO. Ethiopian update sheet on blood safety. 2015; Available from: http://www.afro.who.int/en/ethiopia/ethiopia.

[2] WHO. Global Status Report on Blood Safety and Availability. 2016. 49 p.

[3] Ethiopia Commences World Blood Donor Day 2014 Celebrations.

[4] WHO. Blood Safety for Regional Office for Africa. 2017.

[5] Tapko JB, Toure B, Sambo LG. Status of Blood safety Report of the 2010 Survey.

[6] FMOH, “Health sector transformation plan-I annual performance report 2016/2017”.

[7] Icek A., “The theory of planned behavior,” Organizational Behavior and Human Decision Processes, vol. 50, pp. 179–211, 1991.

[8] Jalalian M, Latiff L, Tajuddin S, Hassan S. Development of A Questionair for Assesing predecting Blood donation Among university students: A Pilot study. 2010;41(3). 20578556

[9] Natan M. B. and Gorkov L., “Investigating the factors affecting blooddonation among Israelis,” International Emergency Nursing, vol. 1, no.3, pp. 1–7, 2010. 10.1016/j.ienj.2010.01.003 21193166

[10] Icek A., “Theory of planned behavour: Implications for an email-based physical activity intervention,” *Psychology of Sport and Exercise Journal*, vol. 9, no. 4, pp. 511–526, 1991. 10.1016/j.psychsport.2007.07.002

[11] Bandura A., “Social cognitive theory of self regulation,” *Journal of Organizational and Human Decision Processes*, vol. 50, pp. 248–287.

[12] Zainie N, Hamid A, Basiruddin R, Hassan N. Factors Influencing the Intention to Donate Blood: The Application of the Factors Influencing the Intention to Donate Blood: The Application of the Theory of Planned Behavior. 2013;(January).

[13] Jimma Blood Bank school based Annual Performance Report,2018 (unpublished).

[14] Mart JD. Intention of future donations: a study of donors versus non-donors. Of J Birtish transfunsfution Med. 2012;(December).

[15] Pule PI, Rachaba B, Gilbert M, Damas M, Habte D. Factors Associated with Intention to Donate Blood: Sociodemographic and Past Experience Variables. 2014;2014. 10.1155/2014/571678 25431742 PMC4238279

[16] Ahuja V, Saluja GP. Assesment of Blood donors perception in A Hospital. 2009;32(2):78–85.

[17] Zainie N, Hamid A, Basiruddin R, Hassan N. The Intention to Donate Blood: An Analysis of Socio-Demographic Determinants. 2013;3(6).

[18] Shibeshi, Na I. Predictors of Intention to Donate Blood among the Eligible Population in Dire Dawa Administration, Eastern Ethiopia: Using the Theory of Planned Behaviour. Blood Disord Transfus. 2018;9(4).

[19] Ossian EN, Eze NC, Chukwu O, Uguru UA UE. Determinants of practice of blood donation among undergraduate students of Ebonyi State University Abakaliki, Southeast Nigeria(2018).

[20] Mramba EL, Ismail IJ. Socio-Economic Determinants of Blood Donation in Tanzania. 2018;4(1):174–82.

[21] Mirutse G, Fisseha G, Abebe L, Birhanu Z. Intention to donate blood among the eligible population in Mekelle City, Northern Ethiopia: Using the theory of planned behavior. 2014;(September).

